# Terminal regions of a protein are a hotspot for low complexity regions and selection

**DOI:** 10.1098/rsob.230439

**Published:** 2024-06-12

**Authors:** Lokdeep Teekas, Sandhya Sharma, Nagarjun Vijay

**Affiliations:** ^1^ Computational Evolutionary Genomics Lab, Department of Biological Sciences, IISER Bhopal, Bhauri, Madhya Pradesh, India

**Keywords:** low complexity regions, positive selection, GC content, repeat diversity, natural selection, evolutionary novelty

## Abstract

Volatile low complexity regions (LCRs) are a novel source of adaptive variation, functional diversification and evolutionary novelty. An interplay of selection and mutation governs the composition and length of low complexity regions. High %GC and mutations provide length variability because of mechanisms like replication slippage. Owing to the complex dynamics between selection and mutation, we need a better understanding of their coexistence. Our findings underscore that positively selected sites (PSS) and low complexity regions prefer the terminal regions of genes, co-occurring in most Tetrapoda clades. We observed that positively selected sites within a gene have position-specific roles. Central-positively selected site genes primarily participate in defence responses, whereas terminal-positively selected site genes exhibit non-specific functions. Low complexity region-containing genes in the Tetrapoda clade exhibit a significantly higher %GC and lower *ω* (d*N*/d*S*: non-synonymous substitution rate/synonymous substitution rate) compared with genes without low complexity regions. This lower *ω* implies that despite providing rapid functional diversity, low complexity region-containing genes are subjected to intense purifying selection. Furthermore, we observe that low complexity regions consistently display ubiquitous prevalence at lower purity levels, but exhibit a preference for specific positions within a gene as the purity of the low complexity region stretch increases, implying a composition-dependent evolutionary role. Our findings collectively contribute to the understanding of how genetic diversity and adaptation are shaped by the interplay of selection and low complexity regions in the Tetrapoda clade.

## 1. Introduction

Proteins do not contain amino acids in equal proportions (5% each for 20 amino acids). Taxonomic dependence yields species-specific amino acid frequencies in the proteome. The regions containing fewer amino acid types than expected, defined by a set of compositionally biased residues and having lower amino acid residue diversity that differs from the average amino acid composition of proteins, are known as low complexity regions (LCRs) [1–5]. LCRs exhibit a variety of amino acid compositions ranging from an aperiodic accumulation of limited amino acids to stretches consisting of a single amino acid residue (homopeptide repeat) [3,6,7]. LCRs are prevalent in eukaryotic proteins, with approximately 18–20% of human proteins featuring at least one amino acid repeat length of five residues or more [3,8,9]. The frequency of LCR-containing proteins is much higher in mammals compared with birds, fishes or amphibians [10]. LCRs have a lower propensity to form structured domains and are much less evolutionarily conserved than globular domains, the most common protein domains [2,11]. Theoretically, the purity of the LCR can vary from >0% to 100% (e.g. a purity of 10% implies only 10 residues of the detected amino acid are present in a stretch of a 100 amino acid LCR, while the purity of 100% implies all the residues in the detected LCR are of the primary residue(s)). Even though the LCRs vary in composition/purity of the stretch, as measured by the ratio of the number of residues of the primary amino acid to the total number of residues in the region, studies are primarily focused on single amino acid (homopeptide) repeats, potentially owing to their ease of search in the protein datasets [3,10,12].

LCRs of various types participate in essential biochemical functions like cell signalling and protein kinases [13], RNA processing [14], transcription regulation [15–17], neurogenesis, DNA repair, recombination [8] and cell adhesion [18]. A series of studies about the consequences of LCRs in neurodegenerative diseases shed light on their functional implications [8,19–21]. Later studies revealed their role not only in diseases but also in evolutionary contexts. Repeats of gene *RUNX2* are linked with facial structure and morphology across vertebrates and are intensively studied in dogs [22,23].

Processes like replication slippage, unequal crossing-over and gene conversion (recombinational mechanisms) make LCRs unstable, leading to length expansion and contraction [13]. This volatility leads to numerous disorders but opens avenues for evolutionary and morphological diversity within a short evolutionary time [23,24]. Variable regions work as ‘tuning knobs’ that create frequent, reversible and site-specific genetic variations, which helps genomes and genes for efficient adaptation [16]. Variable LCRs also participate in immunological pathways. Leucine-rich repeats are a key structural component of toll-like receptors in animals' innate immunity and adaptive immunity in jawless fishes [25–28]. Moreover, LCRs evolve more rapidly than the remaining gene sequence and thus promote evolutionary novelty [29,30]. LCRs also generate polymorphisms within species and play an equally relevant role in microevolution [29,31,32]. In an interesting study on the Great Pyrenees dog, the authors found an association between the polydactyly of the bilateral rear first digit and deletion within a P/Q repeat in the *ALX4* gene [22].

Although amino acid simple repeats are a subset of LCRs, the mechanisms of origin and maintenance vary. The ‘slippage’ model is one of the possible mechanisms for the emergence of amino acid repeats [33]. This model posits that errors occurring during DNA replication or repair can trigger DNA polymerases or other repair enzymes to slip, leading to the insertion or deletion of a few nucleotides. Such events can alter a gene’s coding region, potentially inducing the expansion or contraction of short nucleotide repeats. Ultimately, these changes may culminate in the emergence of amino acid repeats within the protein sequence [13]. The abovementioned mechanism will give rise to homogenous DNA sequence repeats due to slippage in triplets in the coding region. However, heterogeneous DNA sequences can contribute to conserved amino acid homopeptide repeats [34,35]. The existence of homopeptide repeats through heterogeneous DNA sequences imply origin mechanisms other than slippage and maintenance by selective forces for the preservation of homopeptide repeats [3,36].

In contrast to homopeptide repeats, most LCRs are maintained by a complex interplay of mutations and selective forces [3]. In a recent study, authors compared LCRs in protein sequences to simulated proteomes and found that repetitive regions that have simple, repeating patterns could have evolved through neutral processes. However, regions that have cryptic, compositionally biased regions could not have evolved through these processes alone. The study suggests that other biological factors and processes are likely at play in the evolution and maintenance of these complex repetitive regions [37].

Conservation of LCRs across species suggests that they are under natural selection and contribute to adaptation [28,38]. Studies also show that LCRs require a minimum length to create a functional impact, and longer LCRs are more prone to disruption of function [38,39]. In an intriguing study on nuclear proteins, researchers found that protein translocation requires a minimum repeat of six histidine residues [38,39]. LCRs are functionally efficient in a medium range, with small-sized repeats being less functional and longer ones forming protein aggregates and reducing function as proposed for the RUNX2 QA repeat [23]. LCR length variability leads to morphological variability, as demonstrated in *ALX4* and *RUNX2* genes [22]. Thus, LCRs generate rapid genetic variability for selection to act on it. This interaction between selection and LCR length variability can give rise to morphological and evolutionary novelty in a short time.

Even though some studies have focused on genome-wide patterns of LCRs across clades [4,12,40], most of the studies either focus on limited gene sets, specific diseases or limited species [23,41,42]. These focused studies provide a detailed mechanism of protein repeat function in particular species or gene sets. Still, they fail to provide a general mechanism for the evolution and expansion of LCRs across clades. Despite the variation in origin, maintenance and selection pressure based on the purity of composition of LCRs, there is a shortage of studies explicitly addressing these aspects. Furthermore, LCRs and selection work in tightly entangled dynamics, but empirical studies that evaluate the overlap of selection and LCRs are scarce.

In this study, we initially investigate the distribution pattern of LCRs as their purity varies across the Tetrapoda clade by generating results spanning a gradient of LCR purity ranging from >0% to 100%, with purity binned at intervals of 10%. However, for our comparative phylogenetic analysis of LCRs and positively selected sites (PSS), we exclusively select LCRs with a purity above 70%. This criterion enables the inclusion of degenerated LCRs across various species while mitigating the risk of generating false positives due to an overabundance and overlap of LCRs and PSS. Subsequently, we examine the prevalence of PSS and the occurrence of LCRs within genes. We observed a consistent abundance pattern of PSS and LCRs in genes across all clades. Our study also reveals that 7 out of the 12 clades exhibit a significant co-occurrence of LCRs and PSS. In two-thirds of the considered clades, the overlap between PSS and LCRs within a gene is higher than what could be expected by chance. Additionally, our analysis reveals that genes containing LCRs exhibit a significantly lower *ω* (d*N*/d*S*: non-synonymous substitution rate/synonymous substitution rate) compared with genes lacking LCRs across all the clades studied (Class (Aves and Amphibia), Order (Primates, Rodentia, Lagomorpha, Chiroptera, Artiodactyla, Perissodactyla, Carnivora, Testudines and Squamata), Infraclass (Marsupialia) and Superorder (Afrotheria)) within the Tetrapoda clade.

## 2. Material and methods

### 2.1. Dataset preparation

In this study, we retrieved orthologous genes using the National Center for Biotechnology Information (NCBI) datasets (electronic supplementary material, supplementary text: §1) command [43] based on a list of human protein-coding genes obtained from BioMart of Ensembl release 105 [44]. In the case of a gene with multiple protein-coding transcripts within a species, the amino acid sequence with a length and sequence most similar across all the species was selected using a custom script as described in electronic supplementary material, supplementary text: §1. We grouped these genes into 13 clades, encompassing 308 species, as presented in electronic supplementary material, table S1. The species are selected to represent a wide range of life forms within the vertebrate subphylum. Additionally, these species are categorized into various taxonomic ranks, including Class (Aves and Amphibia), Order (Primates, Rodentia, Lagomorpha, Chiroptera, Artiodactyla, Perissodactyla, Carnivora, Testudines and Squamata), Infraclass (Marsupialia) and Superorder (Afrotheria), based on their shared morphology, common ancestry and similar environmental conditions. Electronic supplementary material, table S2 contains a comprehensive list of protein-coding genes used in this study, including each species’ gene and amino acid accession number. We excluded long intergenic non-coding (LINCs), mitochondrial and read-through genes from the 18 407 human protein-coding genes, resulting in 17 940 genes. We identified LCRs with >70% purity in 5768 genes (electronic supplementary material, table S3; LCR purity: number of residues of the primary amino acid in the LCR/total number of residues in that LCR). In non-avian clades, we used the species tree from TimeTree v.4 [45], while the tree for birds was obtained from the BirdTree website [46].

### 2.2. Protein LCR identification

We used fLPS 2.0 [47] to identify stretches of amino acid LCRs. We selected LCRs based on the following criteria: (i) the region should be longer than three amino acids [48], (ii) the stretch should not contain X and (iii) the stretch should not contain more than five unique amino acids in the composition. We investigated the distribution and abundance patterns of LCRs across a composition purity gradient. However, we restricted our analyses of co-occurrence or overlap of LCRs and PSS to those LCRs that exhibited more than 70% purity. The combinations of multi-amino acid LCRs are grouped to only one combination (e.g. ILA, LIA, AIL, IAL, LAI and ALI are grouped to AIL) to facilitate the comparison of orthologous LCRs across species within each clade (electronic supplementary material, supplementary text: §2). The above criteria enhance the identification of orthologous LCRs and minimize artefacts. We provide a list of LCR-containing genes (5768) with more than 70% purity threshold in electronic supplementary material, table S3.

### 2.3. Comparison of orthologous sequences

We removed the coding sequences without a complete open reading frame (ORF), i.e. if they have a premature stop codon, absence of start codon, presence of a non-nucleotide character (any character other than A, T, G or C) or the sequence length is not a multiple of 3. Subsequently, we removed the gene sequences with less than three species in a clade. Strikingly, upon implementing these filtering criteria, the Lagomorpha clade was entirely eliminated from consideration (making the subsequent list comprising 12 clades), rendering it excluded from subsequent molecular evolutionary analyses. We used the GUIDANCE [49] program with MUSCLE [50] aligner with 100 iterations for multiple sequence codon alignment of the orthologous gene sequences and selected the output of full sequence alignment for further analysis (electronic supplementary material, supplementary text: §3). The coordinates of LCRs identified by fLPS 2.0 in the amino acid sequences are mapped to their respective aligned locations in the coding sequence to identify the orthologous LCRs across species in a clade using a custom script (electronic supplementary material, supplementary text: §4). The positional overlap of repeats of different species in the alignment is considered an indicator of orthologous repeats (electronic supplementary material, supplementary text: §4).

### 2.4. Analyses and visualization

We pruned the species trees according to each gene sequence file using the ‘ape’ package in R. We used the site model (M7 and M8) of PAML [51] to identify PSS. A likelihood ratio test was conducted on the compared models to identify signatures of site-specific positive selection. The Bayes empirical Bayes (BEB) method was used to calculate the posterior probabilities of each detected codon under positive selection. Sites with posterior probability >0.95 are considered under significant positive selection [51]. We calculated lineage-specific *ω* (d*N*/d*S*) by implementing the ‘free-ratio’ model (Model = 1) in the PAML package.

### 2.5. Positional preference of LCRs in a gene

To analyse the occurrence frequency of homopolymeric LCRs consisting of a single type of amino acid, we calculated their abundance per unit length of the gene sequence. Specifically, we extracted all LCRs composed of a specific amino acid from all clades and determined their mid-point position to the normalized gene length. We divided the normalized LCR positions into 20 equal-sized bins. We then calculated the proportion of LCRs present in each bin and expected a proportion of approximately 5% in each bin under a completely random amino acid distribution. To identify the potential overabundance or underabundance of LCRs in each bin, we set thresholds of 8% and 2%, respectively. Moreover, we analysed the distribution of each amino acid LCR across the purity gradient to gain insight into the changes in selection.

Additionally, we tested the robustness of the positional preference by LCRs (>70% purity) by varying parameters in the fLPS 2.0 program (−*t* [0.0001–0.1], −*m* [5–15] and −*M* [500–1000] parameters) on a smaller dataset. We assessed the robustness of LCR detection by fLPS 2.0 by calculating the proportion of the same LCRs detected under different parameters in a pairwise manner (electronic supplementary material, supplementary text: §4).

### 2.6. Co-occurrence of PSS and LCR

Using Fisher’s test, we investigated the co-occurrence and overlap patterns between LCR and PSS at three different scales. Specifically, we examined the overlap between LCRs and PSS at a gene-wise and clade-wise level and evaluated their co-occurrence at the clade level (Fisher’s test, *p* < 0.05 for significance). To perform these analyses, we used the fisher function in R to assess co-occurrence and the bedtools fisher tool to examine overlap, considering the coordinates of the LCRs and PSS. We performed all the necessary analyses and visualizations using R [52]. Moreover, to examine the impact of alignment coverage on the overlap between PSS and LCRs, we analysed the alignments of 12 genes representing various clades.

## 3. Results

### 3.1. LCRs show a heterogeneous distribution with purity

**Figure 1.**
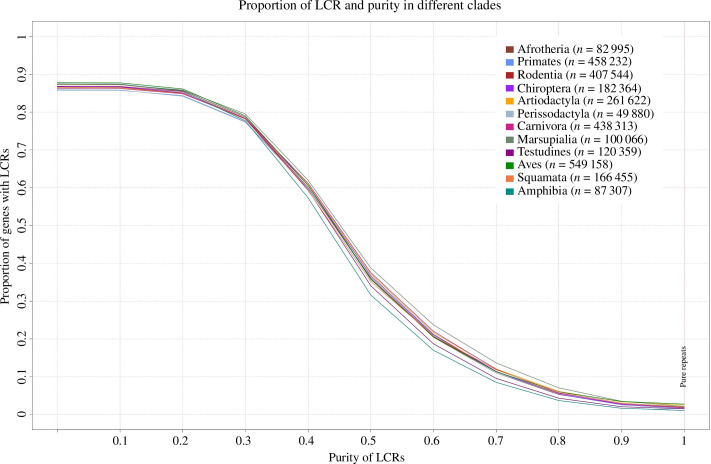
Proportion of LCRs along the purity gradient in different clades. The purity gradient ranges from >0% to 100%. The plot displays a decrease in number of protein-coding genes containing LCRs as the purity of LCRs increase. The numbers in brackets represent the number of LCRs detected in each clade.

We detected ~85% of protein-coding genes to have at least one LCR in all the clades (figure 1). The percentage of LCR-containing genes shows a gradual decline with increasing purity of the LCR composition, with ~9–15% at 70% purity and only ~1–3% at 100% purity. For our comparative phylogenetic analysis of LCRs and PSS, we selectively analysed LCRs with a purity greater than 70% to include degenerated LCRs across various species while reducing the likelihood of false positives arising from an overabundance and overlap of LCRs and PSS.

### 3.2. Protein LCRs show variable lengths across clades

**Figure 2.**
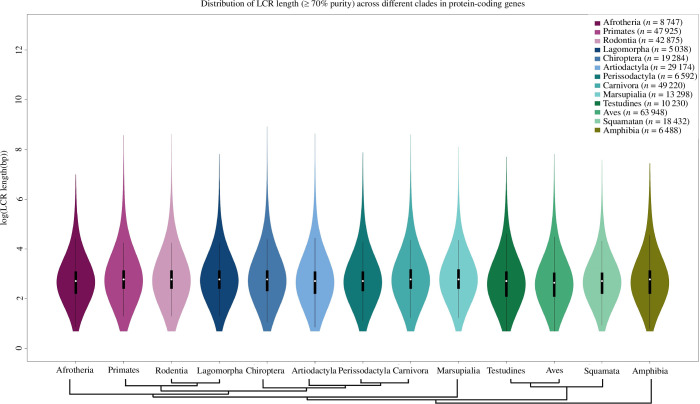
Length distribution of protein LCRs across clades. The violin plot shows the amino acid LCR length distribution in each clade, and the plot width represents the density of LCR length in that class interval. Most LCRs have a length in the lower range, while some are exceptionally large.

Protein repeats can have varying length distribution according to clades and purity. In order to examine the possible divergence in the length distribution of LCRs among diverse clades and levels of purity, we visualized the length distribution for each clade, using a purity gradient of 10%. The resulting figures, presented in both figure 2 and electronic supplementary materials, figures S1–S10, enabled us to carefully scrutinize the possible variations in the skewness of the length distribution for each clade and purity level to gain further insight into the potential clade- and purity-specific impacts on the distribution of LCR lengths (electronic supplementary material, figures S11–S23). Our study demonstrates that low-purity LCRs (>0–60% purity) exhibit larger length variation across different clades. However, the distribution of length becomes more constrained at higher purity levels. Specifically, when the purity level is at or above 70%, the majority of LCRs in all clades are found in the lower range of the length distribution, and only a few regions are exceptionally long (>2 kb). The mean length of LCRs at ≥70% purity across clades varies between 63 and 72 bp. The exceptionally large length LCRs can be an artefact as their orthologues are scarcely present.

### 3.3. LCRs participate in a variety of biological processes

Studies highlighting the major pathways and processes of protein LCRs are scarce and are limited to specific genes [24,40,53]. To evaluate the major pathways and biological processes of protein LCRs, we used gene enrichment analyses using ShinyGO 0.76 server [54] (electronic supplementary material, table S4). The analysis identified enrichment in biological processes related to morphogenesis, regulation of transcription by RNA, neurogenesis and embryo development (electronic supplementary material, figure S24). The cellular component analysis highlights enrichment for the RNA polymerase transcription regulator complex, basement membrane, endoplasmic reticulum (ER) lumen, chromatin and components related to neurons (electronic supplementary material, figure S25). The molecular function is enriched mainly for molecular binding activities (histone, DNA, RNA, chromatin and protein kinase binding) (electronic supplementary material, figure S26). Similarly, the enrichment analyses of PSS-containing genes showed enrichment for biological processes like ion transport and neuron development and differentiation, cellular components like basement membrane and receptor complex, and molecular functions like protein tyrosine kinase and transferase activities (electronic supplementary materials, figures S27–S29 and table S5).

We extended our study to examine position-specific enrichment of pathways for PSS and LCR-containing genes (electronic supplementary material, figures S30–S37 and table S6). We performed the analyses by classifying the PSS and LCR genes into terminal and central classes. Central-PSS genes are enriched only for glycosaminoglycan binding (electronic supplementary material, figure S36), while terminal-PSS genes are mainly for ATP and nucleotide binding (electronic supplementary material, figure S37). The biological processes of central-PSS genes are primarily associated with defence responses (electronic supplementary material, figure S34). Additionally, central-PSS genes exhibit enrichment in components such as collagen-containing extracellular matrix and secretory granules (electronic supplementary material, figure S35). Interestingly, gene ontology (GO) enrichment analyses for terminal-PSS genes showed non-significant results for biological processes and cellular components despite having a large abundance of genes. We can attribute the non-significance of the results to the fact that when the background and foreground gene sets become very similar for GO enrichment, the results become non-significant.

### 3.4. LCR diversity varies across clades

We employed the species–area relationship curve as a conceptual framework to investigate the detection rate of unique LCRs relative to the total number of LCRs in each clade. The slope of the curve represents the rate of change in LCR richness with an increase in the number of LCRs. A steeper slope indicates a faster increase in the number of unique LCRs with the increasing number of LCRs, while a flatter slope indicates a slower increase in LCR richness. We calculated the slope of richness for each clade (electronic supplementary material, figures S38–S52). Furthermore, we also calculated the richness and slope of richness species-wise for each clade and compared the distribution (electronic supplementary material, figures S53–S58). Aves show a high variation in the richness of LCRs and the slope of the curve between species. Moreover, Aves have the highest LCR (>70% purity) richness compared with other clades, while Lagomorpha and Perissodactyla exhibit a low richness (electronic supplementary material, figure S57). After normalizing the LCR richness to the number of initial sequences, the richness across all the clades is comparable (electronic supplementary material, figure S58).

**Figure 3.**
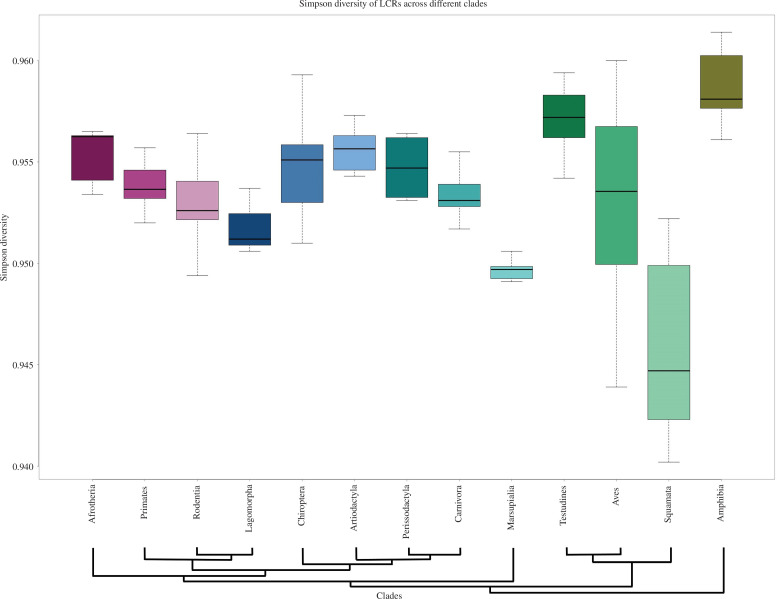
Diversity of LCRs across clades. The boxplot shows the amino acid LCRs diversity of each species in each clade. We calculate the diversity for each species using Simpson’s index of diversity. Simpson’s diversity index takes into account the type and abundance of LCRs. The homogeneous abundance of LCRs leads to a high diversity index, while an unequal abundance of different LCRs leads to a lower diversity index. The Amphibia clade shows an overall higher diversity, while the Squamata clade shows lower diversity of amino acid LCRs.

To overcome the effect of an unequal number of species and the number of complete ORFs in clades, we use the Shannon diversity index (*H*) and Simpson’s diversity index (*D*). Simpson’s diversity index considers richness (the number of unique LCR types defined by the set of amino acid residues present) and abundance (the proportion of each LCR type). If all LCR types have the same abundance, the *D* will be 1, while the overabundance of limited LCR types will lead to a lower *D* value (close to 0). We compare the distribution of species-specific *D* across all the clades (figure 3). Our result depicts that *D* remains very high across all the clades (>0.94). The overall diversity is highest in Amphibia while lowest in the Squamata clade. The Shannon–Wiener diversity index also shows a similar pattern of LCR diversity distribution (electronic supplementary material, figures S59–S63). Similarly, we calculated richness, the slope of the curve and diversity indices for LCRs of 100% purity and found a similar pattern (electronic supplementary material, figures S64–S68).

### 3.5. Clades show variable LCR abundance

**Figure 4.**
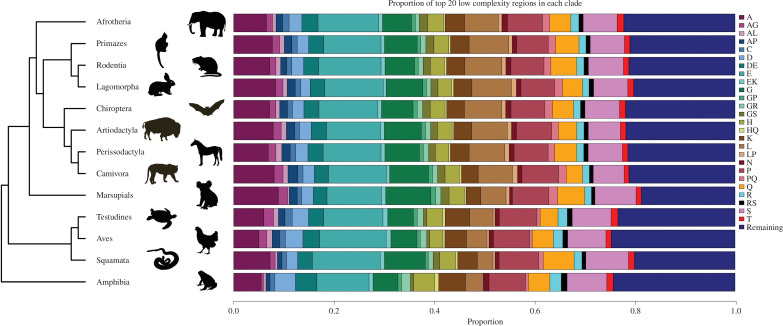
The proportion of the top 20 amino acid LCRs in each clade. The stacked barplot shows the proportion of the top 20 amino acid LCRs across all the clades along the phylogeny. Each colour in the figure represents a different amino acid LCR. The deep blue colour shows the summation of an abundance of all the remaining LCRs in the respective clade.

Factors like clade-specific codon usage, recombination slippage or selection pressure can lead to a varying abundance of LCR types. A high proportion of a particular amino acid LCR in a clade will imply bias towards that amino acid in mechanisms related to the origin or maintenance of LCRs in that clade. We selected each clade’s 20 most abundant LCRs and compared their proportion (figure 4). We observed that clades' most abundant LCR types vary slightly (the top 20 LCRs are not the same across all the clades). For instance, the proportion of the AG LCR is very low in the Amphibia clade but has a high proportion in other clades. However, the proportion for most LCRs remains the same.

### 3.6. LCRs exhibit amino acid-specific positional preference in a gene

Previous studies have emphasized a positional bias for amino acid repeats, where those containing poly-L, V, Q, H, N, C, A and G prefer the N-terminal, while those with poly-S, K, I and F favour the C-terminal [12,42,55,56]. These studies highlight an association between the type of amino acid and biological function but primarily focus on single amino acid repeats. To gain a more comprehensive understanding of the selection constraints acting on LCRs within a biological context, we examined their positional preferences across a purity gradient ranging from 0% to 100%, with 10% intervals, as illustrated in electronic supplementary material, figures S69–S298. Moreover, to investigate taxon-specific preferences, we extended our study in a clade-specific manner along the same purity gradient (electronic supplementary material, figures S299–S441).

Interestingly, LCRs of amino acids A, G, L and R exhibit a pronounced preference for the beginning of the gene, even for compositions of close to 0% and higher purity. Additionally, nine amino acid LCRs (A, V, R, Q, P, L, G, F and C) exhibit an overabundance at the N-terminus of genes, while two residues (D and E) show overabundance at the C-terminus. Furthermore, three residues (Y, N and M) show overabundance at both termini at 70% purity. The distribution of leucine (L) amino acid shows an overabundance at the terminals of the gene sequence while avoiding the middle region (electronic supplementary material, figure S174). It is worth noting that none of the clades exhibit an overall preference for either terminal of the gene up to an LCR purity of 50%. However, for LCR compositions of 50% or higher, all clades prefer the N-terminal of the gene (electronic supplementary material, figures S288–S441).

### 3.7. Genes with LCRs face stronger purifying selection

Previous studies report the GC composition of genes containing repeats to be significantly higher than those without repeats [12,29,40]. These studies were limited to specific organisms, specifically defined gene sets or amino acid tandem repeats. We compared the %GC of all the available protein-coding genes with and without LCRs (>70% purity) in the Tetrapoda clade. We find that the %GC of LCR-containing genes is significantly higher than those without LCRs (Wilcoxon test, *p* < 0.05 in all the comparisons; electronic supplementary material, figure S442).

**Figure 5.**
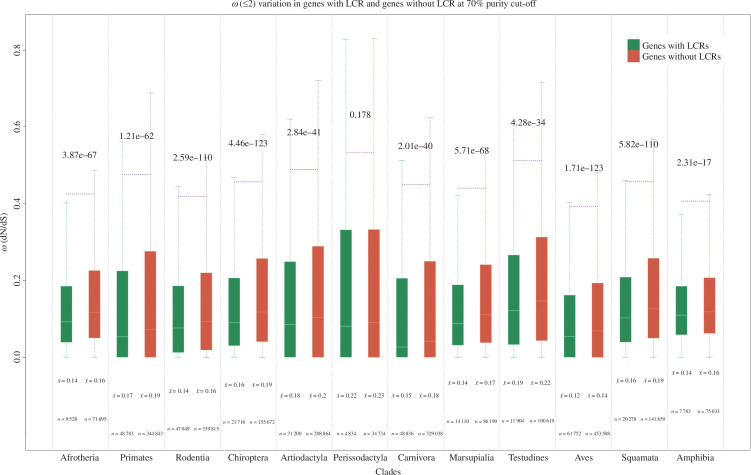
Distribution of *ω* (d*N*/d*S*) in proteins with LCRs and without LCRs. We calculated each lineage’s *ω* (d*N*/d*S*) using the free-ratio model of PAML and subsetted it according to the presence of LCR or absence. The boxplot in green and red colour shows the *ω* distribution of protein sequences with LCRs and protein sequences without LCRs, respectively. The boxplots are visualized with *ω* ≤ 2 and without showing the outliers. The distributions of *ω* are compared using the Wilcoxon test in R in a clade-wise manner. The values above the boxplots represent the *p*-value of the distributions compared. The ‘*x*-bar’ below the boxplot represents the mean *ω* for that distribution, and ‘*n*’ represents the number of lineages used to plot the distribution.

Furthermore, we compared the distribution of *ω* (d*N*/d*S*) between genes with and without LCRs (>70% purity) after filtering out all the *ω* values greater than 2. Filtering out the values greater than 2 is necessary as sometimes PAML reports *ω* values as high as 999, potentially biasing the results. Our finding reveals that LCR-containing genes have significantly lower *ω* distribution than genes without LCRs across all the clades (Wilcoxon test, *p* < 0.05; figure 5). Most of the *ω* values are smaller than 1, implying purifying selection on the majority of the genes. The median and mean of *ω* in genes with LCRs are lower than those without LCRs. The result suggests that genes with LCRs face more substantial purifying selection than others. To rule out the possibility of sampling bias, we compared the distribution with all the *ω* values at different purity cut-offs of LCRs. The inference remains consistent (electronic supplementary material, figures S443–S461).

### 3.8. LCRs and positive selection prefer similar positions in a gene

We calculated the proportion of LCRs and PSS in 10 bins clade-wise in three purity classes of LCR composition, all LCRs, LCRs of >70% purity and LCRs of 100% purity, on a normalized gene length (figure 6 and electronic supplementary material, figures S462–S499). Both LCRs and PSS show a higher abundance at the terminals of the gene while keeping a low abundance in the remaining bins. Furthermore, we examined the distribution patterns of the three aforementioned LCR purity classes in each clade (electronic supplementary material, figures S500–S513) to gain insight into changes in LCR distribution within genes. Our analysis indicates that while all LCRs show a relatively uniform distribution, those with >70% and 100% purity prefer the terminal regions in all clades. To rule out the possibility of alignment coverage bias in the inference of results, we compared the distribution with alignment coverage (electronic supplementary material, figures S514–S539). Alignment coverage shows a negative trend and correlation compared with positive sites and LCRs abundance distribution.

**Figure 6.**
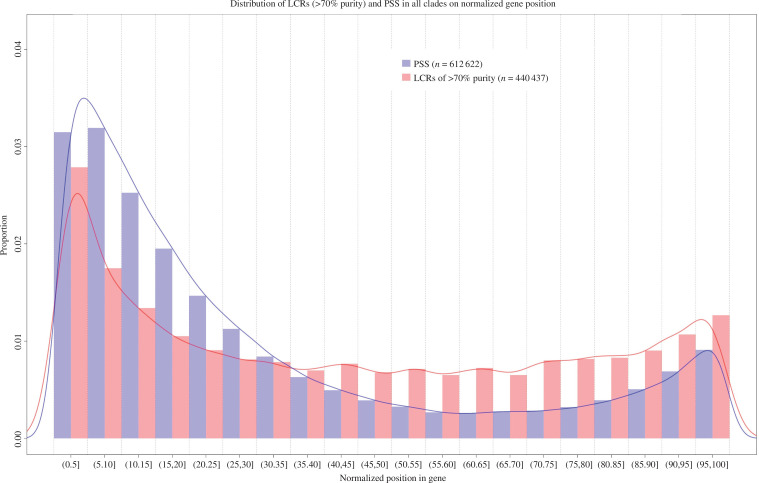
Positive selection and LCRs prefer similar regions in a gene. The overlapping histogram shows the abundance of PSS and LCRs mid-position along the normalized gene length. Selection and LCR show a preference towards the terminal regions of a gene.

### 3.9. Genes exhibit co-occurrence of LCRs and PSS

Given the similarity in distribution patterns of LCRs (>70% purity) and PSS within protein-coding genes, we explored the potential co-occurrence and overlap between the two. We analysed the distribution and patterns of LCRs and PSS within genes across three levels: gene-wise overlap, clade-wise overlap and clade-wise co-occurrence. This comprehensive approach allowed us to understand better how these two features interact within genes. A total of 501 unique genes show a significant overlap (*p* < 0.05; Fisher’s exact test using bedtools fisher) of PSS and LCRs in the Tetrapoda clade, while 363 unique genes exhibit an avoidance of overlap (electronic supplementary material, table S7). Interestingly, 8 of the 12 clades studied significantly favour PSS in LCRs (*p* < 0.05; Fisher’s exact test using bedtools fisher; electronic supplementary material, table S8). Three clades show non-significant results, while Afrotheria significantly avoids PSS in LCRs. Furthermore, our analysis of the co-occurrence of PSS and LCRs showed significant (*p* < 0.05; Fisher’s exact test) co-occurrence in seven clades, except for Perissodactyla, where we observed mutual exclusion (electronic supplementary material, figures S540–S551 and table S9).

We also tested the robustness of the results using a smaller dataset by varying the parameters of fLPS 2.0 (the program implemented to detect LCRs) for the positional preference of LCRs in a normalized gene position. Interestingly, the positional preference for LCRs (>70% purity) shows high robustness even with varying fLPS 2.0 parameters (electronic supplementary material, figures S552–S589) and limited dataset size.

From the visualization of alignments of genes with PSS within LCR, we observed that PSS within LCRs displayed adequate alignment coverage (electronic supplementary material, figures S590–S601). However, the terminal regions of LCRs exhibited low-alignment coverage, attributable to variations in length across different species. This observation aligns with findings from a previous study [57].

## 4. Discussion

LCRs of proteins exhibit a notable proclivity for inducing rapid adaptive alterations within a relatively short evolutionary time, in contrast to the longer-term evolutionary epistasis [3,23]. Additionally, LCRs play a pivotal role in facilitating site-specific, frequent and reversible mutations, serving as an adept tuning mechanism for genes and genomes to promote efficient adaptation [16]. Research has proposed a ‘Goldilocks’ range of lengths, within which variation in LCR length yields non-deleterious functional consequences. When LCRs are excessively short, their functional impact is minimal, whereas regions exceeding a critical length lead to protein aggregation, thereby impeding functionality. LCRs falling within this optimal range confer functional diversity without detriment [23]. The overwhelming evidence about the functional consequences of length variation suggests LCRs face stronger purifying selection above a critical length; an expansion may favour smaller LCRs, and the variation in length is nearly neutral within the critical limit [13]. The length instability and composition volatility in LCRs furnish a foundation for natural selection to operate effectively. Given the close interplay between selection and LCR polymorphism, it is imperative to comprehend their distinct signatures across evolutionary clades.

Moreover, although distinct mechanisms govern the origin and maintenance of homopeptide sequences and other LCRs, studies elucidating the shifts in their abundance, length, positional preferences, as well as the strength of selection acting upon them remain scarce. Considering the prevalence of LCRs with diverse purity compositions within the Tetrapoda clade, the importance of composition in the context of selection is indisputable. Additionally, it is imperative to study the shift in distribution patterns and abundance of LCR along the purity gradient to have a holistic view of the change in the interplay of LCR, purity and selection in the rapid adaptation of species in a shorter evolutionary time.

The abundance pattern of LCRs along a purity gradient can potentially provide insights into the selective constraints and biological significance of these regions. A high prevalence of LCRs may suggest a nearly neutral distribution in an evolutionary context, whereas a limited fraction of conserved LCRs exhibiting very high purity may indicate functional essentiality. Our analysis revealed that LCRs are markedly abundant at lower purity levels (>80% genes with at least one LCR of >30% purity), but only a small subset of LCRs exhibit high purity (<5% with 100% purity) across the entire Tetrapoda clade. This observation implies that LCRs with very high purity are either of recent evolutionary origin or may potentially have functional implications, while the low-purity LCRs could be remnants of degenerated LCRs. Notably, approximately 80% of protein-coding genes contain at least one LCR with a purity of 30% or higher. This finding underscores that the occurrence of LCRs within protein-coding regions is more common than previously assumed, but their persistence is subject to a complex interplay of various evolutionary constraints.

LCRs are significant contributors to the rapid emergence of evolutionary novelty, primarily due to their characteristic length polymorphism. These regions operate within specific length ranges to facilitate functional diversification, as elucidated by Newton & Pask [23]. Remarkably, our analysis reveals that the length distribution of LCRs remains consistent for all purity gradients across all Tetrapoda clades. Very distinct length distribution of orthologous LCRs across clades could imply functional diversification in that clade. Selective constraints play a critical role in governing the length and purity of the majority of LCRs, with smaller regions prevailing more frequently. It is essential to note that the abnormal expansion of LCR length beyond a critical threshold can potentially yield detrimental consequences. A similar length distribution pattern across the clades for all purity composition suggests an equivalent selective constraint of ‘Goldilocks’ range irrespective of purity composition. Notably, in exceptional instances among Primates, LCR length attains unusually large proportions, a phenomenon not observed in other clades or species. We cannot rule out the possibility of annotation artefacts for such LCR-containing genes.

The number of species included in various clades has a direct impact on the identification of unique LCRs within each clade. To assess the relationship between the number of unique LCRs and the number of gene sequences, we employ the species–area relationship curve, a concept borrowed from ecological studies [58]. In this context, we treat the number of gene sequences as analogous to the ‘area’ and the number of detected LCRs as akin to the ‘number of species’. This approach allows us to compare the detectability of LCRs across various clades. Across most clades, the resulting distribution falls within a range of 5.4–5.6. This implies that, under similar conditions of gene sequence detection, each clade exhibits a nearly equal number of unique LCRs. This finding underscores the universal importance of LCRs in different Tetrapoda clades. Given the variations in annotation levels and the number of species within these clades, we also calculate Simpson’s and Shannon–Wiener’s diversity indices to evaluate the LCR community structure [59,60]. Both diversity indices consider both richness (the presence of unique LCRs) and evenness (the uniform distribution of LCRs). The Simpson’s diversity index (*D*), which ranges from 0 to 1, where 0 signifies no diversity and 1 represents maximum diversity, falls within the range of 0.94–0.96 across all species within all clades. Similarly, the Shannon’s diversity index (*H*), ranging from 0 to *n* (where *n* is any positive number), with 0 indicating no diversity, consistently ranges between 3.6 and 3.8. The uniformity in these diversity indices suggests a similar composition of LCRs across all clades, regardless of the number of genes considered for each species. These findings underscore the integral role played by LCRs in various functions across diverse species within the Tetrapoda clade. Their indispensable contribution aids in maintaining repeat diversity across different clades.

We conducted a comparative analysis of the proportions of the 20 most abundant LCRs within each clade. Notably, among all amino acid LCR types, alanine, glycine and proline consistently emerge as the most prevalent types across all clades. Specific amino acid LCRs have been previously associated with distinct functional implications in various biological processes [13]. The conspicuous presence of particular LCR types within specific processes is hypothesized to play a contributory role in the evolutionary development of those specific pathways [13]. In the context of mammalian proteins, genes containing alanine repeats are notably overrepresented in functions related to DNA binding and RNA binding [12]. Alanine-rich regions play a prominent role in neurogenesis, signalling and development [31,61–63]. Therefore, the high abundance of alanine LCRs across Tetrapoda clades is in alignment with these established functional associations. Interestingly, alanine, proline and glycine LCRs have more evolutionary effects than other LCRs. PolyP, polyA and polyG repeats are known to modulate protein–protein interactions and regulate transcription [64–67]. Additionally, proline-rich regions have been found to prevent aggregate formation when positioned on the C-terminal side of glutamine repeats, with this effect diminishing if PolyP is relocated to the N-terminal side of the PolyQ, as indicated by Bhattacharyya *et al*. [68]. The essential roles of these specific LCRs, coupled with their functional influence on other LCRs, provide a plausible explanation for their higher prevalence within the Tetrapoda clade.

In addition to the distinct functional roles associated with amino acid-specific LCRs, it is noteworthy that specific LCRs exhibit preferences for particular positions within genes, underscoring their position-dependent functions [69]. Notably, leucine LCRs tend to favour the N- and C-terminals of genes while largely avoiding the mid-portions. Most LCRs exhibit a preference for the gene’s start over other positions, with the exception of D, E, K and Y LCRs. Conversely, aspartate, glutamate, lysine and tyrosine LCRs display a predilection for the end terminal over other positions. Intriguingly, serine LCRs do not demonstrate any specific positional preference. Moreover, C, F, I, M, N, V, W and Y LCRs are consistently found in low abundance across all clades, with W LCRs being the scarcest. This scarcity is highlighted by a prior study that, while investigating the abundance and distribution of amino acid repeats, failed to detect any tryptophan (W) repeats [56]; we observed only a minimal presence of W LCRs. The proportion and position preferences of other amino acid repeats align with the findings of a prior study [56]. It is worth noting that most leucine repeats located at the N-terminal of genes serve signal peptide functions and are secreted [56]. Furthermore, our finding emphasizes the consistent prevalence of LCRs at lower purity levels while simultaneously revealing a positional preference within genes as the purity of the LCR stretch increases. Collectively, these observations provide compelling evidence of the position- and composition-dependent functions of amino acid LCRs.

A noteworthy observation pertaining to the overall abundance of LCRs within genes indicates a distinct preference for terminal regions over the central region. Specifically, the N-terminal region is more densely populated with LCRs than the C-terminal region, suggesting that the 5′ end of the gene exhibits greater dynamism in LCR generation [12]. Furthermore, the tendency of LCRs to avoid the middle region of a gene may represent an evolutionary adaptation aimed at circumventing the globular regions and potential domain misfolding [12,56]. These findings underscore the region-dependent nature of selective constraints within genes, with certain regions displaying a higher tolerance for diversity than others. The substantial presence of LCRs within the malleable regions of a gene may facilitate functional diversification. Intriguingly, a similar trend is observed concerning PSS along genes across the Tetrapoda clade. Both LCRs and PSS exhibit a pronounced preference for the initial region of a gene, with the central region hosting the smallest proportion.

Genes containing PSS exhibit a notable enrichment in functions related to neurogenesis, transcription regulation and morphogenesis. These PSS genes are also enriched in various molecular functions, including tyrosine kinase activity, transporter functions and ATP binding. Intriguingly, we find that genes containing LCR and PSS genes share a similar enrichment in biological processes, hinting at the equal significance of positive selection and LCRs in maintaining and diversifying these processes. Furthermore, it is worth noting that PSS genes positioned in the middle of the gene (mid-PSS) and those located at the gene’s terminals (terminal-PSS) demonstrate distinct enrichments in molecular functions. Terminal-PSS genes are primarily associated with functions related to ATP and nucleotide binding, whereas central-PSS genes display an enrichment for glycosaminoglycan binding. Notably, among the two categories of enriched functions, only seven genes are common to both, with the majority being unique to each function. The prevalence of unique genes in both categories suggests a position-dependent role of selection based on specific functional attributes.

A previous study by Lenz *et al.* identified an intriguing relationship between the distance from LCRs, the average substitution rate per site and the proportion of genes containing gaps in the alignment at each site. In addition, their analysis of d*N*/d*S* ratios revealed a negative correlation between the proportion of sites subject to negative selection and the distance from LCRs [57], i.e. the proportion of sites experiencing negative selection decreases with distance from the LCR. Furthermore, a separate investigation highlighted that sites demonstrating evidence of positive selection tend to cluster near the boundaries of LCRs, with a higher concentration of such sites observed on the N-terminal side of the LCR boundary in comparison to the C-terminal side. Specifically, approximately half of these sites were situated within the first 26 residues nearest to the LCR boundary, while 95% of them fell within a range of 201 residues. Importantly, this clustering pattern persisted even when focusing exclusively on the most strongly selected sites. This skewness in distribution might be influenced by the direction of transcription rather than inherent protein structural factors [42]. We also considered the potential impact of poor alignment coverage in terminal regions on the detection of PSS. Notably, the program used for detecting PSS, PAML, treats regions with suboptimal alignment as missing data [70]. Regions characterized by variable lengths are more likely to have suboptimal alignment quality and may consequently be excluded from the analysis. Our findings indicate a negative trend and a correlation between alignment coverage and the presence of both PSS and LCRs. This suggests that LCRs often exhibit suboptimal alignment due to their variable length. However, it’s important to note that the reduced alignment coverage does not necessarily result in the detection of a lower number of PSS within the region. Apart from our finding, a study detected a similar trend of a faster evolutionary rate at the termini of a protein sequence even after removing poorly aligned regions [71]. This further enhances the presence of malleable regions at the termini of protein sequences to harbour evolutionarily innovative opportunities.

The comparison of the GC content revealed that LCR-containing genes have a significantly higher %GC than genes without LCRs across the whole Tetrapoda clade (Wilcoxon test; *p* < 0.05). The result is consistent with previously published studies on specific gene sets [12,40]. A high %GC in LCR-containing genes suggests that GC-rich regions are prone to LCR formation. GC-rich codons primarily encode LCRs, increasing the overall %GC [12]. A high GC content has far-reaching implications, as it increases mutation and recombination rates and influences gene length due to DNA polymerase slippage [72,73]. Replication slippage is one of the central mechanisms for the emergence and maintenance of protein LCRs [15,74]. These findings collectively suggest that a higher GC content fosters a predisposition to LCRs, elevates mutation and recombination rates, and heightens the occurrence of DNA polymerase slippage. The emergence of LCRs, in turn, increases the overall GC content of the gene, as GC-rich codons mainly encode LCRs. So, protein LCRs and GC content work in positive feedback dynamics where the existence of one can promote the emergence and maintenance of the other if no other biological constraint exists.

Natural selection plays a crucial role in the emergence and maintenance of LCRs [28,38]. A newly emerged LCR faces relaxed and positive selection, which promotes rapid diversification. However, a functionally crucial fixed LCR is under purifying selection [28]. Moreover, purifying selection keeps the LCRs composition pure, while relaxed selection leads to compositional amalgamation [22]. We observe that LCR-containing genes have significantly lower *ω* (d*N*/d*S*) than genes without LCRs for all the purity gradients across a majority of the clades of Tetrapoda. This finding is consistent with a previous finding on a smaller dataset with only the comparison of d*N* [13]. Furthermore, LCR-containing genes displaying a significantly lower *ω* than the genes without LCRs across all the purity gradients in the majority of the clades implies that specific genes have a higher propensity for the origin of LCRs and are governed by significantly stronger purifying selection. Despite the intensified purifying selection acting upon genes with LCRs, these regions exhibit enrichment for PSS. They serve as reservoirs of functional diversity and evolutionary novelty, indicating an interplay between selection and mutation in governing LCR functionality.

## 5. Conclusion

Protein LCRs play a vital role in generating rapid functional diversity and evolutionary novelty, with their specific characteristics, positions and lengths influencing their biological functions. The interplay between mutation and selection governs the features of LCRs, and it is noteworthy that pure amino acid repeats and regions with compositional bias have distinct origins and maintenance mechanisms. Our observations indicate that approximately 80% of protein-coding genes encompass at least one LCR with a purity exceeding 30%. Additionally, we observe a significant association between PSS and LCR, with both preferring similar positions within genes and frequently co-occurring in various Tetrapoda clades. LCRs are notably enriched in specific biological pathways and exhibit consistent diversity across different Tetrapoda clades. These LCRs are predominantly present in functionally significant genes, subject to rigorous purifying selection and characterized by a higher %GC compared with genes without LCRs. Furthermore, we observe that PSS and LCRs tend to favour the terminal regions of genes over the central region. Genes containing central- and terminal-PSS, as well as central- and terminal-LCRs, are associated with distinct biological processes. Notably, our study emphasizes that the terminal regions of genes are more adaptable to accommodate variations and are host to dynamic LCRs and PSS. Additionally, we note that LCRs consistently exhibit widespread prevalence at lower purity levels but display a preference for specific positions within a gene as the purity of the LCR stretch increases. This suggests a composition-dependent function of LCRs. Our study enhances our understanding of the intricate dynamics between natural selection and LCRs, shedding light on the mechanisms governing the emergence and persistence of protein LCRs while also deepening our comprehension of their role in protein diversification and the dynamic processes shaping their presence in biological systems.

## 6. Limitations and future directions

This study included 13 clades comprising more than 300 species with more than 18 000 protein-coding genes to rule out sampling bias. However, this does not take care of all the confounding factors. Species have different genome assembly and annotation levels. Furthermore, the number of species between clades varied because of the unavailability of proper annotation in some clades. A clade comprising only a few species with good annotation will reflect the results of only those species and not the whole clade. Clade-specific results can potentially be affected by the number and combination of species selected for the study.

We used fLPS 2.0 for LCR sequence identification and filtered out sequences with less than 70% LCR composition, less than four amino acid lengths and more than four amino acid combinations. Even though we tested the robustness of the results using a smaller dataset by varying the parameters of detection in fLPS 2.0, the results can still potentially vary if the program to identify LCRs is changed. Moreover, we used the MUSCLE aligner with 100 bootstraps for sequence alignment. Using a different aligner can potentially affect the alignment and thus, the results associated with the aligned files. Most PSS are in low-alignment coverage regions. This result needs further analysis to rule out the effect of alignment.

Despite the potential confounding factors, the results provided open a new avenue for further exploration of the evergrowing field of protein LCR evolution. Our results explore only the Tetrapoda clade. Analysing other clades can help us understand the mechanism and evolution of protein LCRs in the genomes of those clades. Furthermore, the orthologous LCRs vary in length between species of the same clade. The impact of LCR length variation on protein structure can allow us to understand their role in diseases in greater detail. Moreover, LCRs are recognized for their overlap with intrinsically disordered regions, playing a pivotal role in protein folding and stability. LCRs demonstrate notable conformational flexibility, lack well-defined secondary structures and are actively engaging in molecular recognition. Notably, LCRs have the potential to bolster tertiary structure stability through interactions with other protein domains. The investigation of LCRs in protein structure and stability remains an active research area. Exploring the impact of LCRs on the dynamics of natural selection, mutation rates and protein stability promises intriguing insights in future studies. Additionally, understanding the involvement of interacting LCR-containing genes in diseases, particularly neurodegenerative disorders, holds promise for improving prognostic capabilities.

## Data Availability

Data are available on Zenodo [75]. Supplementary material is available online [76].
